# Quantitative indices for an intracranial aneurysm and subarachnoid hemorrhage in early childhood: a case report

**DOI:** 10.1186/s12883-022-03022-4

**Published:** 2022-12-19

**Authors:** Kenichi Tetsuhara, Noriyuki Kaku, Koichi Arimura, Yasunari Sakai, Shouichi Ohga

**Affiliations:** 1 Department of Pediatrics, Graduate School of Medical Sciences, Kyushu University, 3-1-1, Maidashi, Higashi-ku, Fukuoka, 812-8582 Japan; 2 Emergency and Critical Care Center, Kyushu University Hospital, 3-1-1, Maidashi, Higashi-ku, Fukuoka, 812-8582 Japan; 3 Present address: Department of Critical Care Medicine, Fukuoka Children’s Hospital, 5-1-1, Kashii-Teriha, Higashi-ku, Fukuoka, 813-0017 Japan; 4 Department of Neurosurgery, Kyushu University, 3-1-1, Maidashi, Higashi-ku, Fukuoka, 812-8582 Japan

**Keywords:** Intracranial aneurysm, Modified Alberta stroke program early CT score (mASPECTS), Outcome, And children

## Abstract

**Background:**

Intracranial aneurysms (ICA) rarely occur in children under 3 years of age. Little is known for neuroimaging parameters that predict survival and clinical outcomes of patients with ICA in early childhood.

**Case presentation:**

A 2-year-old girl showed intracranial hemorrhage due to a rupture of aneurysm at the middle cerebral artery. Quantitative measurements of ischemic damages on the head computed tomography (CT) marked an extremely low score of 2 points with modified Alberta Stroke Program Early CT Score (mASPECTS). She died 15 days after admission. In publications from 2021 to 2022, we found 21 children who were under 3 years of age at onset of ICA. None of them died, but two of three patients who had mASPECTS scores 0–8 showed developmental delay and/or epilepsy as neurological complications.

**Conclusion:**

Early CT findings are applicable for predicting survival and neurological outcomes of young children with intracranial hemorrhage.

**Supplementary Information:**

The online version contains supplementary material available at 10.1186/s12883-022-03022-4.

## Background

Intracranial aneurysm (ICA) is a rare condition in children under 3 years of age [1, 2]. Infections, post-traumatic, and specific genetic conditions are more frequently associated with ICA and ICA-related brain hemorrhages in childhood than those in adults [3]. However, only a few reports have demonstrated details in neuroimaging features and clinical outcomes of patients with ICA in early childhood [4, 5]. Two neuroimaging parameters, simplified gray matter attenuation-to-white matter attenuation ratio (sGWR) and modified Alberta stroke program early CT score (mASPECTS), are known to be useful for quantitatively analyzing parenchymal damages of the brain in children with cardiac arrest [6]. We thus asked whether these scoring systems might also provide critical values for the outcome of ICA in early childhood.

We herein report a young child who had a rupture of ICA at the middle cerebral artery (MCA) and characterize the neuroimaging feature of this patient in comparison with previously reported children under age 3 years.

## Case presentation

**Fig. 1:**
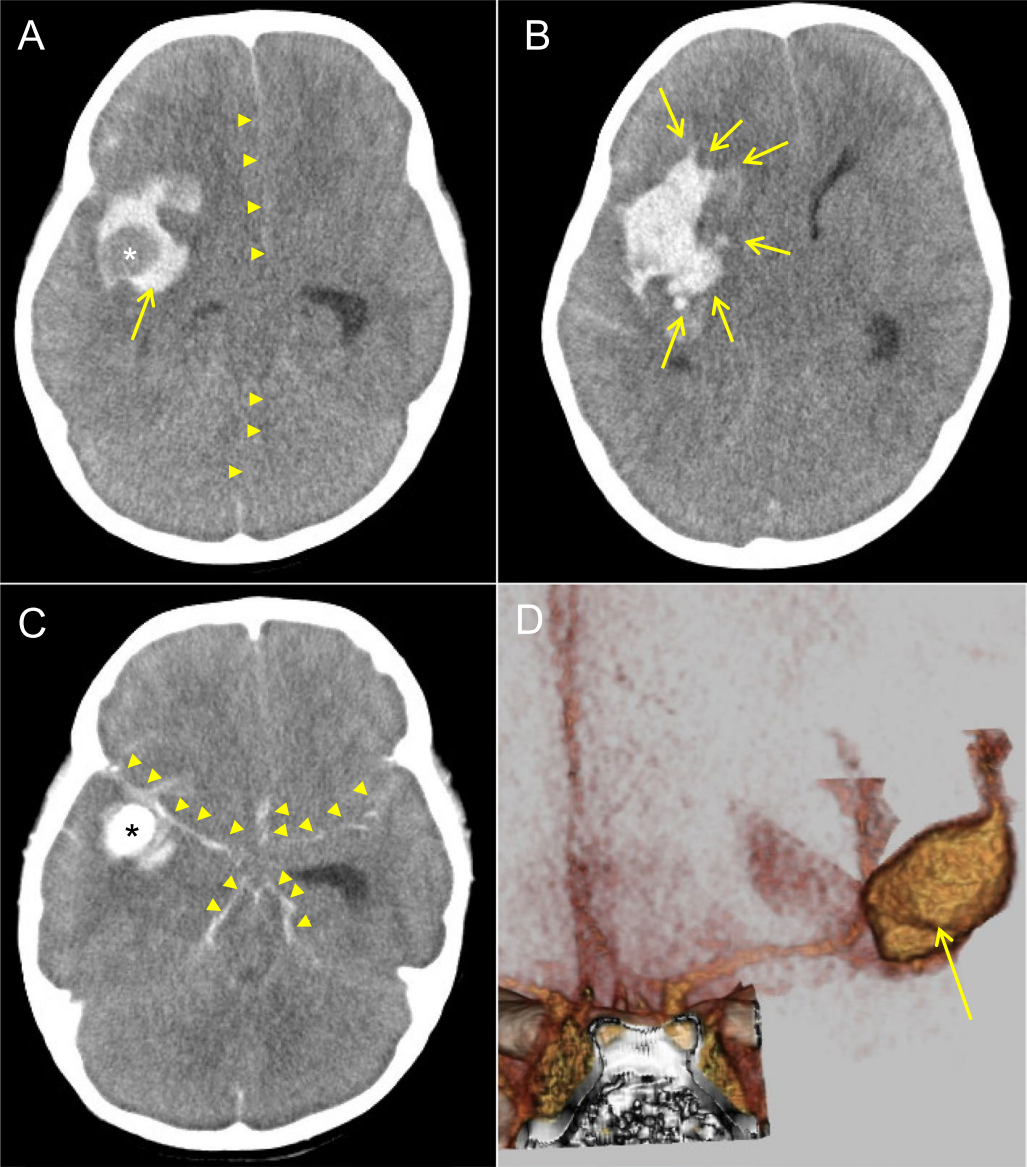
Plain and contrast-enhanced CT scans of the aneurysm in the present case. **A** A plain CT on admission in the present case. Arrow indicates the massive hemorrhage and the devoid of hemorrhagic signal (asterisk) in the right MCA region. Arrowheads indicate the prominent midline shift to the left hemisphere. **B** A plain CT on admission. The hemorrhagic lesion was extended to the surrounding parenchyma involving the caudate and lentiform nuclei (arrows). **C** A contrast CT shows the presence of an aneurysm located at the right MCA region (asterisk) and subarachnoid hemorrhage (arrowheads). **D** A stereographic reconstitution of the contrast head CT depicting the fusiform structure of MCA aneurysm (arrow: 21 × 13 × 12 mm in size)

A 2-year-and-8-month-old girl was referred to the previous hospital because of altered consciousness. A head computed tomography (CT) indicated the urgent neurosurgical intervention for the intracranial hemorrhage with a midline shift. On arrival to our hospital, spontaneous breathing was absent, and Glasgow Coma Scale was evaluated to be E1V1M1. The contrast-enhanced CT in our hospital confirmed the hemorrhage extending to the subarachnoid space and disclosed an aneurysm of 21 × 13 × 12 mm at the right MCA (Fig. 1). We applied quantitative measurements of the sGWR and mASPECTS (range 0–24) for her neuroimaging data (Fig. S1). The modified sGWR scored 1.13, while mASPECTS gained only 2 points, both suggestive of poor prognosis [6]. Although decompressive surgery was immediately performed, her systemic conditions became unstable with uncontrollable pulmonary edema. She died on the 15th day after admission. The panel sequencing for *COL3A1*, *FBN1*, *TGFBR1*, *TGFBR2*, and *RNF213* excluded the diagnosis of Ehlers-Danlos syndrome, Marfan syndrome, Loeys-Dietz syndrome, and Moyamoya disease. Blood culture was negative throughout the treatment course. Systemic imaging studies excluded malformation of great arteries, renal cysts, and tumors.

Through the literature search from 2021 to April 2022 in PUBMED (https://pubmed.ncbi.nlm.nih.gov/), we found 103 publication records with search terms, “intracranial”, “aneurysms” and “pediatric”. Among them, 21 patients were reportedly under 3 years of age (12 females; 1 months to 2 years) at onsets (Table 1) [4, 5, 7–12]. Although variable degrees of neurological deficits were left, none of them died or failed to receive neurosurgical operations for their critical conditions. Thus, no clinical or neuro-imaging parameters were identified to predict the unfavorable outcome (death). Based on the neuroimaging data (*n* = 10), however, we estimated mASPECTS scores. We classified them into the three groups: L (low, mASPECTS 0–8), M (moderate, 9–16) and H (high, 17–24) (Table 1). Two (67%) of the three patients who belonged to the L group showed profound complications of developmental delay and/or epilepsy as neurological sequelae. On the other hand, two (29%) of seven patients with M and H scores showed hemiplegia. These data suggested that mASPECTS scores were useful for predicting postsurgical outcomes of ICA.

**Table 1:** Summary of patients with intracranial aneurysms less than 3 years of age (reports in 2021–2022)

No	Patient ID	Age at onset^1^	Location, morphology	Etiology	mASPECTS^2^	Outcome^3^	Year	Ref
1	Xu-1	1 m	L-cavernous, saccular	Unknown	–	mRS 0	2021	5
2	De Aguiar-1	1.5 m	R-MCA 10 mm, saccular	Idiopathic	–	–	2022	8
3	Clarke-7	2 m	R-MCA 11 mm, saccular	Idiopathic	L	DD, EP	2022	4
4	Clarke-3	3 m	R-MCA 9 mm, fusiform	Idiopathic	L	DD	2022	4
5	Clarke-6	3 m	L-PCA 16 mm, saccular	Idiopathic	–	DD	2022	4
6	Komuński-1	5 m	R-ICA, saccular	Idiopathic	M	–	2021	10
7	Clarke-5	6 m	R-MCA 2 mm, saccular	Idiopathic	–	Hemiparesis	2022	4
8	Clarke-9	6 m	L-MCA 14 mm, saccular	Idiopathic	–	Hemiparesis, VD	2022	4
9	Clarke-8	7 m	R-ICA 5 mm, saccular	Idiopathic	–	Hydrocephalus	2022	4
10	Sombo-1	8 m	Proximal basilar 8 mm, fusiform	Post-infectious	L	–	2021	12
11	Xu-2	9 m	L-MCA, pseudo	Unknown	–	mRS 2	2021	5
12	Barch-1	10 m	R-MCA, multiple, fusiform	Idiopathic	H	–	2021	7
13	Saraf-1	11 m	L-MCA, multi-lobular, dissecting	Post-traumatic	H	Hemiplegic gait	2021	11
14	Clarke-10	12 m	R-PCA 10 mm, saccular	Gaucher disease	–	Hemiparesis	2022	4
15	Xu-3	12 m	L-MCA	Unknown	M	mRS 1	2021	5
16	Clarke-1	1 y 2 m	L-MCA 4 mm, saccular	Idiopathic	–	Minor spasticity	2022	4
17	Clarke-4	1 y 5 m	R-MCA 18 mm, saccular	Idiopathic	H	Hemiparesis	2022	4
18	Xu-4	1 y 9 m	R-MCA, saccular	Unknown	M	mRS 1	2021	5
19	Xu-5	1 y 10 m	L-cavernous, giant	Unknown	–	mRS 0	2021	5
20	Clarke-2	1 y 10 m	R-ACA 3 mm	Post-traumatic	–	No deficit	2022	4
21	Demartini-1	2 y	R-ICA 5 mm, saccular	Post-traumatic	M	Asymptomatic	2021	9
22	Present case	2 y 8 m	R-MCA 21 mm	Idiopathic	L, 2	Died on day 15		

^1^Age represents years (y) and months (m)

^2^The mASPECTS scores were estimated and classified into the following three groups according to the presented images: L (low, 0–8), M (moderate, 9–16) and H (high, 17–24). -, no image available

^3^Comorbidities and neurological outcomes include epilepsy (EP) and measurements in modified Rankin scale (mRS)

-, No complication or normal development; DD, developmental delay; VD, visual defects; mASPECTS, modified Alberta Stroke Program Early CT Score; Ref, references

## Discussion

ICA in pediatric age accounts for 10–15% of the whole patient populations [13]. Patients under 3 years of age are extremely rare in prevalence [14, 15]. While it is difficult to perform angiography for infants and young children during the critical period, pediatric patients with successful outcomes have been increasingly reported in recent years [3–5, 8, 11, 12]. Nevertheless, data have been less extensively analyzed for young children with unfavorable clinical courses. For this reason, the present case underscores the diagnostic value of neuroimaging findings for accurately detecting the ICA in early childhood.

In our previous study, lower scores (sGWR < 1.14 and mASPECTS < 20) were correlated with worse outcomes [6]. The present case showed low values of sGWR (1.13) and mASPECTS (2) on admission. Although further studies are required, these data may compensate insufficient prognostic values for survival and neurological outcomes of pediatric patients with intracranial hemorrhage.

Different patho-mechanisms have been considered to be involved in the development of ICAs in childhood and adults [1, 2, 4]. Childhood-onset ICAs are more frequently associated with trauma, infection, and particular genetic backgrounds than adult-onset ICAs [16]. In our patient, either the family information, past history, laboratory data or genetic analysis did not support evidence for common causes of ICAs. Thus, the patient was etiologically classified into the group of “idiopathic” ICA [1].

In conclusion, contrast-enhanced CT is a useful modality not only for detecting the source of hemorrhage, but also for predicting survival and neurological outcomes of young children with massive ICA. Accumulating clinical and quantitative neuroimaging data will further dissect critical findings for both groups of children with successful and unfavorable outcomes.

## Supplementary Information

**Additional file 1.**

## Data Availability

The datasets supporting the conclusions of this article are all available in this manuscript.

## Abbreviations

ICA: Intracranial aneurysm
CT: Computed tomography
mASPECTS: Modified Alberta Stroke Program Early CT Score
sGWR: Simplified gray matter attenuation-to-white matter attenuation ratio
MCA: Middle cerebral artery
