# Supplementary material for: Quantitative indices for an intracranial aneurysm and subarachnoid hemorrhage in early childhood: a case report
Source: BMC Neurol. 2022 Dec 19;22:488. doi: 10.1186/s12883-022-03022-4 (PMC9761937; doi:10.1186/s12883-022-03022-4)
Supplement: Supplementary file 1 — Additional file 1. [file 12883_2022_3022_MOESM1_ESM.docx]

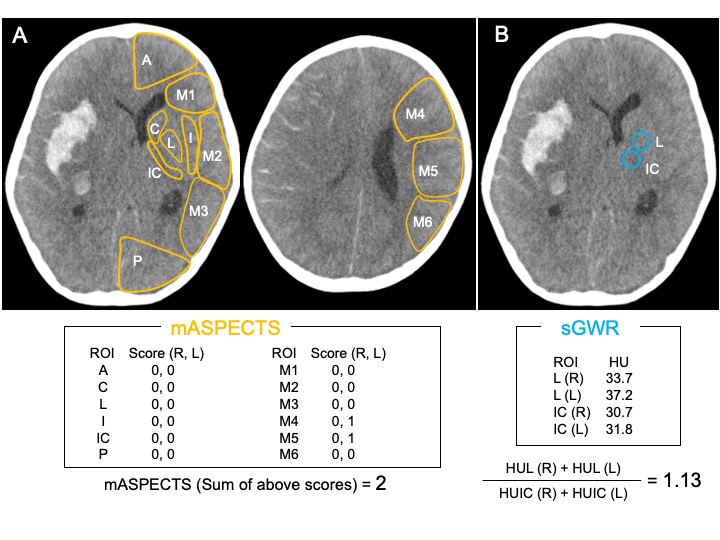


**Figure S1.** Contrast-enhanced CT on admission for the quantitative measurements of sGWR and mASPECTS.

1. Orange lines indicate 12 regions of interest (ROI) for the left hemisphere. Of 12 ROIs per hemisphere, damaged regions were defined as 0, while each intact region was counted as 1. Modified ASPECTS were the sum of each score of ROIs. This patient had only two regions that were considered intact (left M4 and M5). Thus, mASPECTS scored 2.
2. Brue lines indicate ROIs for simplified GWR. The CT values were defined as 33.7 (right lentiform, L), 37.2 (left L), 30.7 (right posterior limb of the internal capsule, IC) and 31.8 (left IC) at the basal ganglia level. Thus, sGWR of this patient was calculated as (33.7 + 37.2)/(30.7 + 31.8) = 1.13.

A: anterior circulation, C: caudate, I: insular ribbon, L: lentiform, M: middle circulation, P: posterior circulation
